# Finite Element Simulation and Multi-Factor Stress Prediction Model for Cement Concrete Pavement Considering Void under Slab

**DOI:** 10.3390/ma13225294

**Published:** 2020-11-23

**Authors:** Bangyi Liu, Yang Zhou, Linhao Gu, Xiaoming Huang

**Affiliations:** 1 School of Transportation, Southeast University, Nanjing 211189, China; 230169539@seu.edu.cn (B.L.); gulinhao@seu.edu.cn (L.G.); 2 School of Materials Science and Engineering, Southeast University, Nanjing 211189, China; tomaszy@seu.edu.cn

**Keywords:** concrete pavement, void underneath the slab, finite element model, predictive function, maximum tensile stress

## Abstract

Uneven support as result of voids beneath concrete slabs can lead to high tensile stresses at the corner of the slab and eventually cause many forms of damage, such as cracking or faulting. Three-dimensional (3D) finite element models of the concrete pavement with void are presented. Mesh convergence analysis was used to determine the element type and mesh size in the model. The accuracy of the model is verified by comparing with the calculation results of the code design standards in China. The reliability of the model is verified by field measurement. The analysis shows that the stresses are more affected at the corner of the slab than at the edge. Impact of void size and void depth at the slab corner on the slab stress are similar, which result in the change of the position of the maximum tensile stress. The maximum tensile stresses do not increase with the increase in the void size for relatively small void size. The maximum tensile stress increases rapidly with the enlargement in the void size when the size is ≥0.4 m. The increments of maximum tensile stress can reach 183.7% when the void size is 1.0 m. The increase in slab thickness can effectively reduce maximum tensile stress. A function is established to calculate the maximum tensile stress of the concrete slab. The function takes into account the void size, the slab thickness and the vehicle load. The reliability of the function was verified by comparing the error between the calculated and simulated results.

## 1. Introduction

Jointed plain concrete pavement (JPCP), known as its significant compressive strength and durability, is designed as one feasible ridged pavement style in those heavy traffic load areas [1]. The surface course, which consists of cement concrete, is supported directly on the treated base course or fully compacted subgrade soil. Since concrete slabs are far more rigid than asphalt surfaces, those common theories for asphalt concrete such as elastic layer theory are no longer suitable. Thin plate theory is advised as one alternative when performing analyses for cement concrete pavements. This theory is based on a previous assumption, which is that the foundation (base course or subgrade soil) is regarded as consistent [2]. This opinion is also mentioned by other researchers [3]. It can adequately simplify the mechanical analysis of pavement response. Nevertheless, one main problem is falls short in illustrating the behaviors when some undetermined but natural outcomes occur. Many studies have found that there are voids beneath the cement slab, which are an un-avoidable damage in the pavement service duration, particularly near the corner or edge of the slab [4,5,6]. The occurrence of voids can result in high tensile stresses at the corner of the slab and eventually cause many forms of potential, such as cracking or faulting [7].

For a period of time, great efforts have been made to understand how the void beneath concrete slabs is generated [8,9]. Inhomogeneous compaction during roadbed construction will lead to uneven support of the base or subgrade, which has a negative impact on the durability of pavement. Rainwater penetrating into the foundation (base or subgrade) through joints and cracks is pumped out under the pressure of vehicle loads, while aggregate particles are flowed away from the foundation [10]. Repeated traffic load can lead to soil consolidation and plastic accumulative deformation in base course [11]. Based on those, the relationship between void and volume loss in foundation was discovered by a series of research [12].

Previous research on the mechanical response of concrete pavements has been conducted by several pioneers, especially Westergaard, who developed closed-form equations to calculate displacement and stress of the slab under loading at the edge, interior and corner [13]. The derivation of equations was based on several grossly restrictive assumptions, which limited the widely used of their original and modified forms. In recent years, many different numerical simulation methods are applied in the analysis of rigid pavement structures, because of the ability to consider complex loads and arbitrary geometries (e.g., void underneath concrete slab) [14,15,16,17]. The finite elements method is the most widely used, as a result of its excellent computational accuracy. Many general-purpose finite element packages have attracted the attention of engineers, such as ABAQUS and ANSYS [18,19,20,21,22]. Besides this, a series of sub-processors were developed in order to satisfy special purpose, such as KENSLABS [23], EverFE [24] and ISLAB2000 [25].

In recent years, most research on concrete pavement mainly focus on the influence of other factors on the response of pavement structure, such as temperature, dowel bar [26,27,28]. Foundation is generally considered consistently uniform in their research. The relationship of void and pavement deflection and detecting techniques of void underneath slabs attract more attention [29]. Some studies analyze the impact of void on pavement stress [30]. Hydrodynamic pressure distribution in the saturated void beneath cement concrete pavement slabs was studied by simulation [31]. It is concluded that surface slab corners are not only the most possible locations for hollow space occurrences, but also critical loading positions that lead to maximum tensile stresses and vertical deflections [32]. Much attention has been paid to the dynamic response of pavement [33,34,35]. Nevertheless, static load instead of pulse load has little influence on the calculation results of concrete pavement [36]. The relationship between void area and stress is not linear, and a larger void area has a greater effect on stress [37]. However, the coupling action of void and traffic load remains un-clear and needs to be analyze quantitatively. Furthermore, the void depth is so large that the slab and base can never contact in the void space under traffic loads in their research. The results of in-situ coring show that the slab is not completely separated from the base course in the early stage of void development. The slab and base course can still contact in the void area under the traffic load. Few studies have considered the effect of void depth on slab stress, such as the change from non-contact to contact between slab and base in void space under traffic load.

The main purpose of this study is to investigate the effect of void on the maximum tensile stress. In this paper, ABAQUS 6.14 is selected due to its excellent simulating ability. The reliability of the model was validated by mesh convergence analysis and comparison with the calculation results of the design standards in China. Two positions, five sizes and many depths of the void were analyzed in the validated FEA (Finite element analysis) model with a single concrete slab. Slab thickness and vehicle load are also considered in the validated model. A stress prediction formula is proposed based on the analysis results. Super computing resources can help to reduce the burden of large problem.

## 2. Materials and Methods

### 2.1. FEA Model Parameters

The analysis model of concrete pavement was worked out in a three-dimensional Cartesian coordinate system and corresponded to a selected motorway pavement in China. Four factors, which are geometric size, mesh, load and material properties, have a great influence on the accuracy of the finite element model. The model consists of two structural layers, which are concrete surface course layer and cement and fly-ash stabilized macadam base course layer. The Winkler foundation is used to simulate the structural layer below the base course layer. There are two methods to build the Winkler foundation in ABAQUS. Several studies use spring elements of type SPRING1 to idealize the subgrade [38]. In this study, the interaction type of Elastic Foundation is used [39].

The surface course of the pavement consists of one concrete slab, which ignores the effect of adjacent slabs on it. Previous studies have indicated that an extended base can effectively decrease the stress of slab, which is more in line with the engineering [40]. Numerous studies have shown that linear elastic constitutive in the model can help to obtain rational results [41], and so it was used in this paper. The three-dimensional finite model characteristics are presented in Table 1.

**Figure 1:**
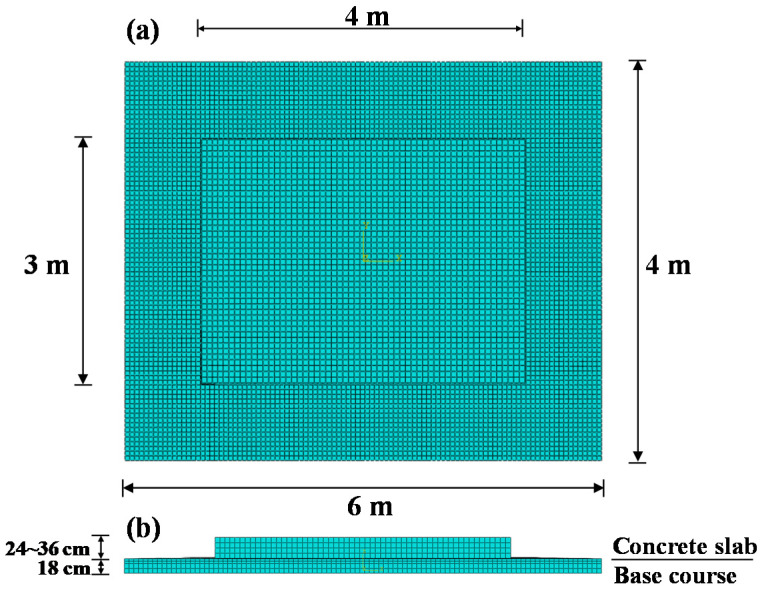
The FEA model of concrete pavement structure: (**a**) plane size of the model and (**b**) vertical size of the model.

Meshing is an important part of an FEA model, so that finer meshes can bring about better results. However, a finer mesh will lead to a sharp increase in the number of elements and nodes in the finite element model, which will result in excessive computational cost. The mesh convergence analysis was used to obtain the finite element type and size, which can ensure the convergence and accuracy of pavement response with a minimum number of elements. The models were carried out by using eight-node incompatible modes linear hexahedra solid elements (C3D8I—concrete slab) [37] and eight-node reduced-integration linear hexahedra solid elements (C3D8R—base course). The FEA model of concrete pavement structure has shown in Figure 1.

The interaction between concrete slab and base course layer was modeled as a contact problem. Hard contact, available in the ABAQUS library, was used in “surface-to-surface” contact mode. The relative slide behavior between the concrete slab and the base course layer was taken into account, but not the sliding friction, i.e., the friction coefficient is 0 [42]. This can help to obtain the most unfavorable stress values in the slab.

**Figure 2:**
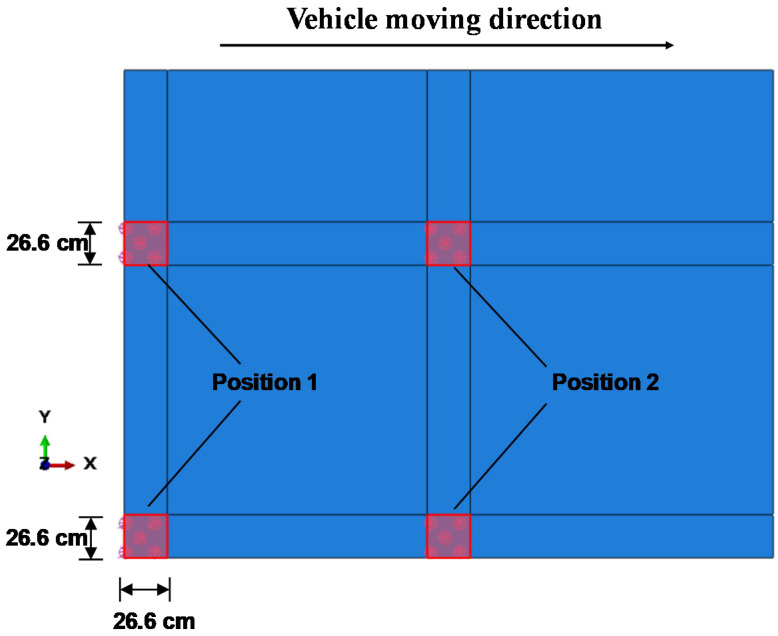
The shape and position of the vehicle load on the slab of FEA model.

The shape of contact areas between wheels and pavement surface is change into a rectangular with the same area. In general, wheel paths of vehicles always change along the transverse direction of the slab. The most unfavorable case is considered in this study. The outermost wheel is at the longitudinal edge of the slab and moves from the corner (position 1 to the middle of the longitudinal edge (position 2) of the slab, as shown in Figure 2. A boundary condition of the fixed placement in the horizontal direction is applied to the base course. All displacements at nodes on all side faces of the concrete slab are free [43].

### 2.2. Void Morphology for Simulation

**Figure 3:**
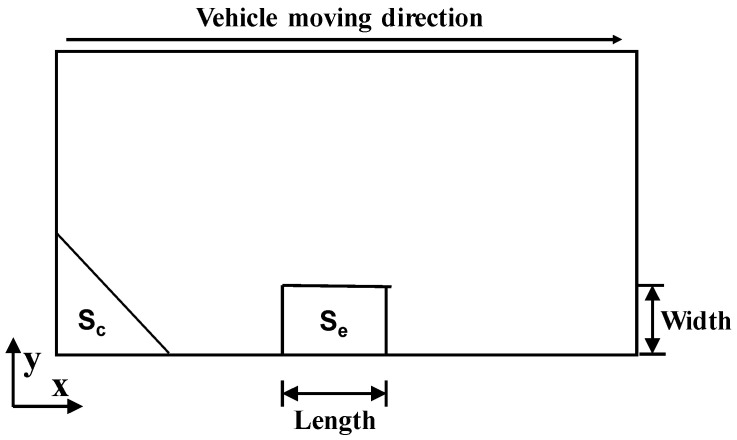
Void morphology of the FEA model.

The horizontal and vertical morphology of void were considered in this study. Previous studies have shown that the horizontal morphology of the void is square at the edge of the slab and triangle or semicircle at the corner of the slab [37]. In Figure 3, the morphology of the void at the corner was an isosceles right triangle. Five side lengths were taken into account for the right-angle side, which are 0.2 m, 0.4 m, 0.6 m, 0.8 m and 1.0 m. While the morphology of the void at the edge was a rectangle. The area of rectangle was equal to that of triangle in order to analyze the influence of void location on the stress of slab. Three length–width ratios of rectangles are considered (2, 1 and 0.5), where length refers to the direction of travel and width refers to the transverse direction of the road.

In this study, the effect of void depth on slab stress was analyzed by varying the height of the void region. The initial height of the void area was 0.2 mm, and increased 0.2 mm each time until the slab stress no longer changes.

### 2.3. Validation of Numerical Models

**Figure 4:**
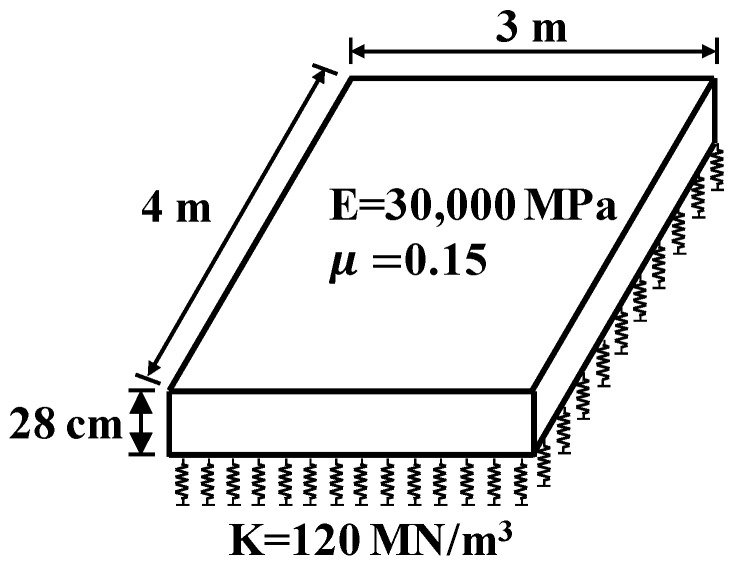
FEA model for convergence analysis.

The degree of mesh fineness and element selections are the most important factors for an accurate evaluation of stresses in a FE analysis. C3D8R elements have been commonly used in past studies to discretize concrete slabs because of their small computational cost. The result of displacement calculation is quite accurate. However, it has a large error in the stress calculations unless the mesh element is divided sufficiently finely. Twenty-node quadratic element (C3D20 or C3D20R) [44] and 27-node quadratic element (C3D27 or C3D27R) [37] can overcome this shortcoming and consequently they are used by some researchers. Nevertheless, 20-node quadratic element cannot be used to analyze contact problems, so it is only suitable for concrete slab on Winkler foundation. It is necessary to rewrite INP file for analysis using 27-node quadratic element, which is not only difficult, but also complex. The FEA model for convergence analysis has shown in Figure 4.

**Figure 5:**
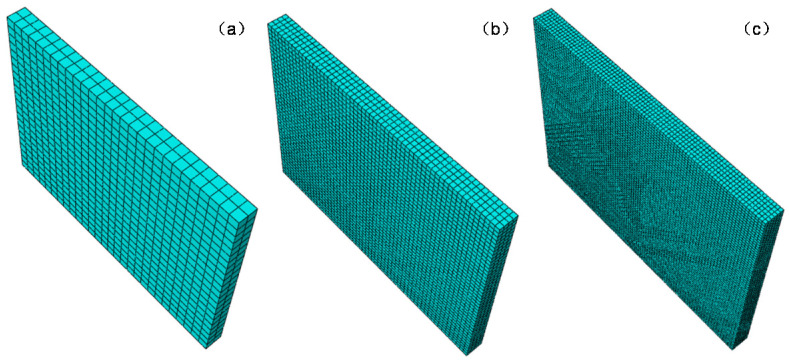
FEA models with different densities: (**a**) h/e = 2; (**b**) h/e = 4 and (**c**) h/e = 6.

The mesh convergence analysis was carried out with concrete slab on Winkler foundation. Five element types (C3D8, C3D8R, C3D8I, C3D20, C3D20R) and three mesh sizes (h/e = 2, 4 and 6) were adopted, which is shown in Figure 5. The mesh size is represented by h/e, where h is the slab thickness and e is the mesh length.

The results of the convergence analysis are presented in Table 2. It is obvious to see that the accuracy of stress results of the linear element (C3D8 and C3D8R) is very poor. Even if the mesh is finely divided, the error rates as high as 20%. Meanwhile, incompatible elements can produce extremely accurate results when h/e = 4. Consequently, the horizontal dimensions of the elements (C3D8I) used for the concrete slab are 7 cm × 7 cm × 7 cm with four layers in the thickness direction.

**Figure 6:**
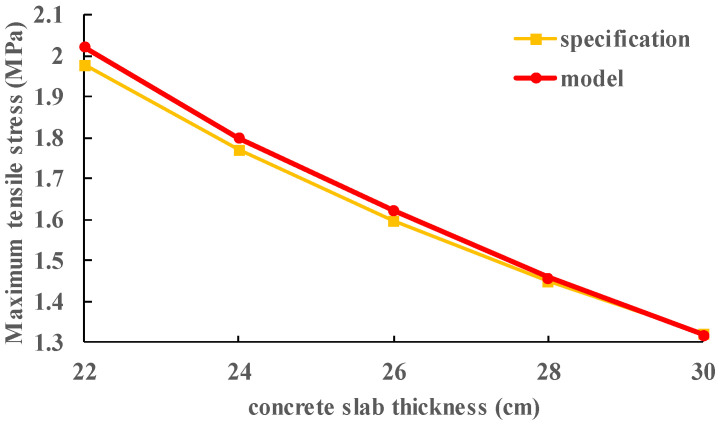
Comparison of results of model and standard.

In order to verify the reliability of the established two-layer model, the calculation results are compared with the calculation results according to the specifications [45], as shown in Figure 6. It can be seen that the calculation results of the model are highly consistent with those of the specification, with an error of less than 3%.

**Figure 7:**
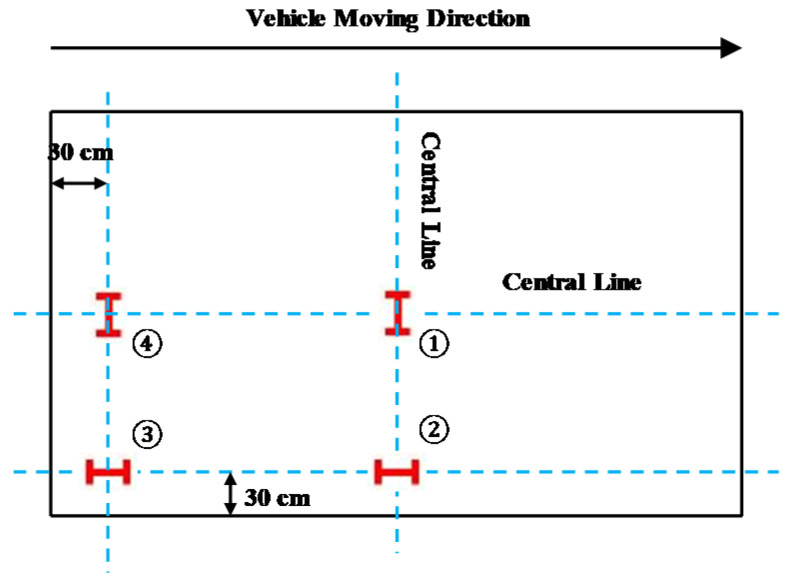
Instrumentation layout.

**Figure 8:**
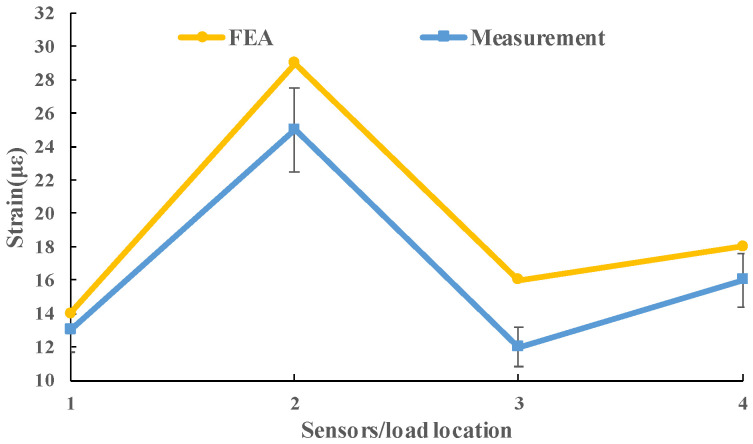
The results of the measurement and simulation.

A filed measurement was conducted to verify the FEA model. The sensors used in the measurement are considered to be able to obtain the strain of the concrete slabs perfectly. The load was applied on each sensor and the data of the corresponding sensor were obtained. Each concrete slab was measured four times. The final results were the average value of total three slabs. Figure 7 shows the instrumentation layout for the filed measurement. The strain sensors were placed at a depth of 3.5 cm from the top (3) or bottom (1,2,4) of the concrete layer. Standard axle load (single wheel) was applied in the four areas (above the sensors). The measurement and simulation results were shown in Figure 8. It can be seen that the calculation results of the model are relatively close to the field measurement results, especially at the slab center. Slight differences can be observed at the slab corner and the slab edge, which may be due to the action of the dowel bar. Considering the variability of pavement and measurement error, an FEA model can produce high precision simulation results.

## 3. Results and Discussion

### 3.1. Impact of Void Size at the Slab Edge

**Figure 9:**
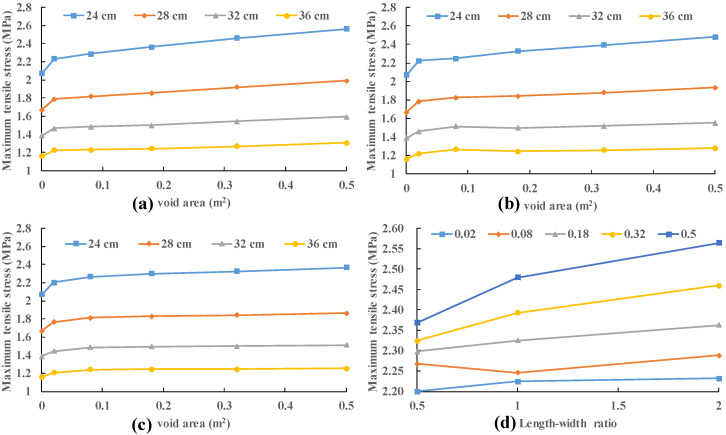
Variation in stress of the slab with various sizes and length–width ratio of void area: (**a**) length–width ratio = 2; (**b**) length–width ratio = 1; (**c**) length–width ratio = 0.5; and (**d**) slab thicknesses = 24 cm.

The void depths are provided as 5 cm to ensure that the concrete slab does not contact with the base course under vehicle load. Five sizes of void area are used: 0.02 m^2^, 0.08 m^2^, 0.18 m^2^, 0.32 m^2^ and 0.5 m^2^. Three length–width ratios (2, 1 and 0.5) are considered for each size. Four slab thicknesses (24 cm, 28 cm, 32 cm and 36 cm) were also analyzed. The used vehicle load was 0.7 MPa, which is also specified in the specifications [45]. The results obtained by applying the vehicle load to the edge of slab (position 2) are presented in Figure 9.

It can be seen that the variation of length–width ratio has little effect on the slab stress. With the increase in void size, the relative difference of slab stress under different length–width ratios also increases. The maximum variation of slab stress under different length–width ratio is less than 2% with a 0.02 m^2^ void area and less than 8% with a 0.5 m^2^ void area. The maximum and minimum increment of stress in different thickness slabs are 11.5% and 4.9% when the void area increases from 0.02 m^2^ to 0.5 m^2^. This increment seems to be very small. Compared to the slab with no void area, the maximum and minimum increment of stress in different thickness slabs are 23.9% and 12.6% with void area 0.5 m^2^. It indicates that the slab stress will increase dramatically when a void area occurs. Nevertheless, slab stress does not change significantly as the void size increases. This may be caused by the change in the stress mode of the concrete slab. The stress mode of the concrete slab in the void area is similar to the bending of the beam supported on three sides, in which the tensile stress at the bottom of the beam is not sensitive to the length of the beam (void area). Consequently, the void at the edge of slab is neglected in the following analysis.

### 3.2. Impact of Void Size at the Slab Corner

**Figure 10:**
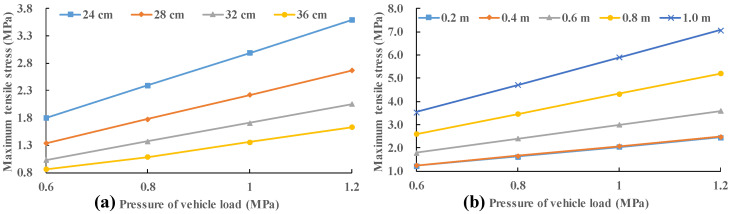
Variation in stress of the slab with various vehicle load: (**a**) void size is 0.6 m; and (**b**) slab thickness is 24 cm.

The consideration of void size (0.2 m, 0.4 m, 0.6 m, 0.8 m and 1.0 m) and slab thickness (24 cm, 28 cm, 32 cm and 36 cm) is consistent with Section 3.1. In addition, the effect of vehicle load on the slab stress is also considered. The pressure of vehicle load is provided as 0.6 MPa, 0.8 MPa, 1.0 MPa and 1.2 MPa. In Figure 10, there is a good linear relationship between the panel stress and the load when the void size is 0.6 m or the panel thickness is 24 cm. In fact, this rule still holds for other void sizes and slab thickness. It is easy to see that when the void size is one meter, the tensile stress of the slab will be very large, even exceeding the tensile strength of concrete. This means that too large a void will lead to larger stress in the slab corner, which will result in the fracture of the slab corner. Since the damage of concrete is not taken into account in the FEA model, the slab stress may exceed the tensile strength of concrete. In the following analysis, the load of 0.8 MPa is taken as an example.

**Figure 11:**
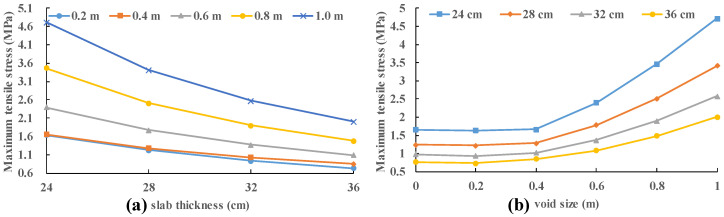
Variation in stress of the slab with slab thickness and void size: (**a**) slab thickness; and (**b**) void size.

In Figure 11, the stress of the slab remains almost constant with the increase in the void size regardless of the slab thickness for relatively small void size (0 m, 0.2 m and 0.4 m). While the stress of the panel increases rapidly with the enlargement of the void size for relatively large void size (0.6 m, 0.8 m and 1.0 m). In order to study this interesting trend, the position of the maximum stress is recorded. When vehicle load (0.7 MPa) moves along the slab (28 cm) edge (position 1 to position 2), the maximum tensile stresses under different load positions are recorded with no void area.

**Figure 12:**
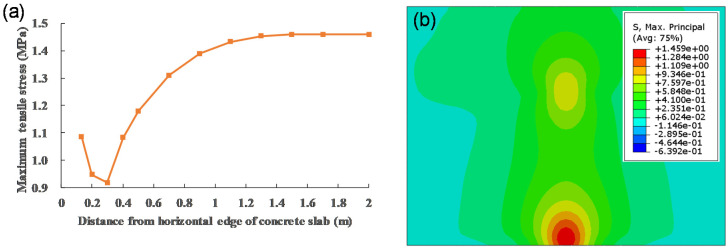
(**a**) Variation in stress of the slab with position of load; and (**b**) Stress nephogram of slab bottom under loading at position.

**Figure 13:**
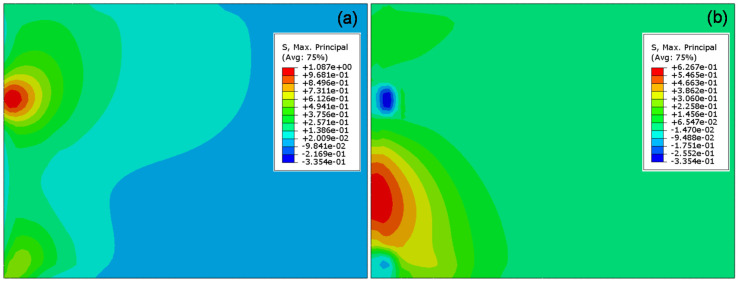
Stress nephogram of slab under loading at position 1: (**a**) the bottom of slab; and (**b**) the top of slab.

As shown in Figure 12, the maximum tensile stress of concrete slab is obtained by applying vehicle load at the edge of slab (position), which is the worst loading position of slab. Maximum tensile stress occurs at the bottom of slab in Figure 13b. When the load is applied to the slab corner, the tensile stress of the slab decreases to 75%. This conclusion is also supported by previous studies [46]. Maximum tensile stress (1.087 MPa) occurs at the bottom of slab under the inner wheel. The tensile stress (0.627 MPa) at the top of slab is 60% of the maximum tensile stress (1.087 MPa).

The maximum tensile stress can still be obtained at the bottom of the slab with 0.2 m void size. The point is that the stress at the bottom of the plate decreases slightly while the stress at the top increases rapidly compared to the stress with no void area. Further calculations show that the most unfavorable load is still at position 2. The maximum tensile stress is obtained at the top of the slab (above the edge of the void) with 0.4 m void size. It means that the stress at the slab top exceeds the stress at the slab bottom. The maximum tensile stress when the load is at the slab corner (position #1) is greater than that when the load is at the slab edge (position 2), which means that the most unfavorable load position is at position 1. Both occurred during the increase in the size of the void from 0.2 m to 0.4 m. In the following analysis, the relatively small void sizes (0.2 m and 0.4 m) will not be considered.

In Figure 11, the stress increases by about 183.7% when the panel thickness is 24 cm with the increase in the void size from 0.4 m to 1.0 m. This value is 166.9% for a slab thickness of 28 cm; 151.6% for a slab thickness of 32 cm; and 135.4% at a slab thickness of 36 cm. As the void size increases, the maximum tensile stress increase more and more rapidly. The increase in slab thickness can effectively reduce the increase in maximum tensile stress.

### 3.3. Impact of Void Depth at the Slab Corner

**Figure 14:**
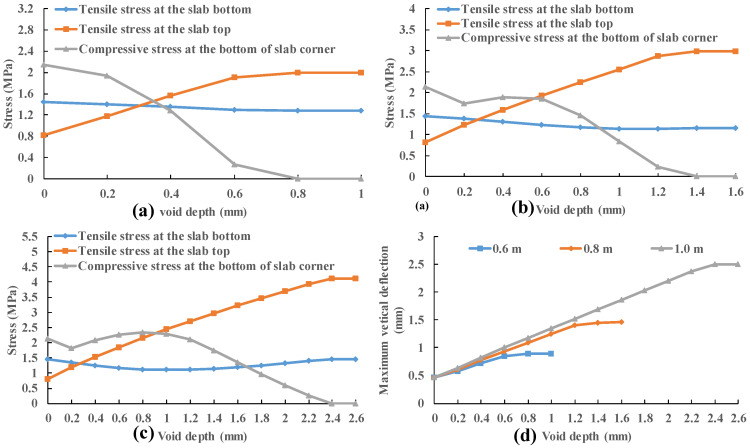
Stress variation of 24 cm thick slab with void depth: (**a**) void size is 0.6 m; (**b**) void size is 0.8 m; (**c**) void size is 1.0 m and (**d**) vertical deflection.

Three void sizes (0.6 m, 0.8 m and 1.0 m) and four slab thicknesses (24 cm, 28 cm, 32 cm and 36 cm) are considered in this section. The initial height of the void area was 0.2 mm and increase 0.2 mm each time until the slab stress no longer changes. The stress of different positions of concrete slab under 0.7 MPa load is recorded. The compressive stress at the bottom of the slab corner is the contact stress between concrete slab and base course. It can be seen that the stresses at the bottom and top of the slab do not change when the compressive stress at the slab corner is reduced to zero in Figure 14. This means that the depth of voiding has been increased sufficiently so that the slab never contacts the base course and the slab stress is independent of the void depth.

**Figure 15:**
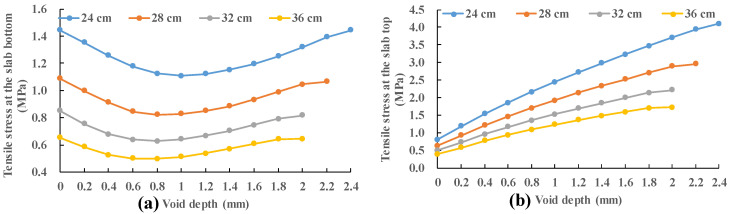
Tensile stress variation of 1.0 m void size with void depth: (**a**) stress at the slab bottom and (**b**) stress at the slab top.

In Figure 15, the stress at the bottom of the slab decreases first and then increases with the increase in the void depth. However, the variation is very small, which is within 20%. The stress on the top of the slab increases with the increase in the void depth until the cement slab no longer contacts the base course. There is approximately a secondary correlation between the slab top stress and void depth. The stress of slab top gradually exceeds the stress of slab bottom when the void depth increases from 0.2 to 0.4 mm. This law is similar to that of the variation of slab stress with the void size.

### 3.4. Regression Analysis of Maximum Tensile Stress

In Section 3.2, the effects of slab thickness, void size and vehicle load on the maximum tensile stress of slab are analyzed. In this section, the function for obtaining the maximum tensile stress through these three factors is presented. Since the most unfavorable load position of the slab is always at the edge of the slab (position 2) when the void size is small (0.2 m), this function only considers the case when the void size is large (0.4 m, 0.6 m, 0.8 m and 1.0 m).

**Figure 16:**
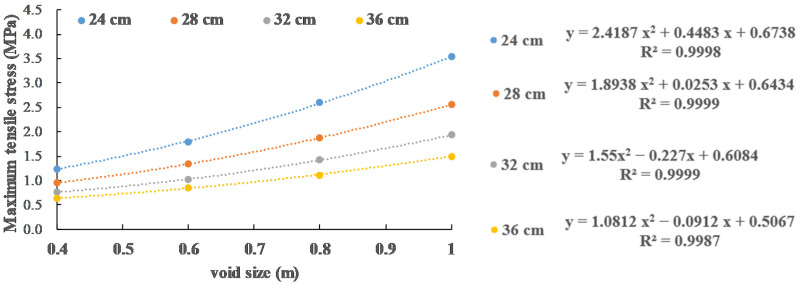
Regression relationship between maximum tensile stress and void size under 0.6 MPa load.

It can be seen from Figure 16 that the error between the curve obtained by quadratic regression and the simulation calculation result is within 2%. When the panel thickness is 24 cm, 28 cm, 32 cm and 36 cm, the relationship between the panel stress function and the void size as the independent variable is as follows:

*σ* = 2.4187 *x*^2^ + 0.4483 *x* + 0.6738 (1)

*σ* = 1.8938 *x*^2^ + 0.0253 *x* + 0.6434 (2)

*σ* = 1.55 *x*^2^ − 0.227 *x* + 0.6084 (3)

**Figure 17:**
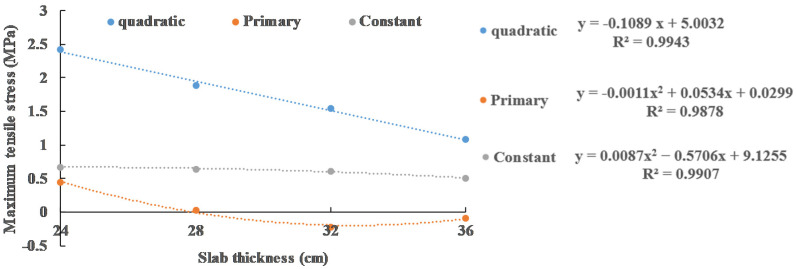
Regression relationship between maximum tensile stress and slab thickness under 0.6 MPa load.

*σ* = 1.0812 *x*^2^ − 0.0912 *x* + 0.5067 (4)

where σ is maximum tensile stress; x is the void size and x≥0.4 m. The quadratic term, primary term and constant term of *x* are regressed, respectively, in Figure 17 and the stress calculation function considering both void size and slab thickness is obtained:

(5) $$\begin{array}{c} \sigma =\left(-0.1089\;h+5.0032\right)x^{2}+\left(0.0087\;h^{2}-0.5706\;h+9.1255\right)x\\ +\left(-0.0011\;h^{2}+0.0534\;h+0.0299\right) \end{array}$$

Five void sizes (0.6 m, 0.7 m, 0.8 m, 0.9 m, 1.0 m) and three panel thicknesses (26 cm, 30 cm, 34 cm) are considered. 15 examples are additionally calculated for validation. The results are presented in Table 3. The maximum error between the results of the formula calculations and the model calculations is 2.36%. The results show that the use of this function to predict slab stress is reliable.

Considering the effect of load size on stress, this function needs to multiply the load adjustment factor. The final function is as follows:

(6) $$\begin{array}{c} \sigma =\frac{P}{85}[\left(-0.1089\;h+5.0032\right)x^{2}+\left(0.0087\;h^{2}-0.5706\;h+9.1255\right)x\\ +\left(-0.0011\;h^{2}+0.0534\;h+0.0299\right)] \end{array}$$

where σ is maximum tensile stress (MPa); x is the void size and x ≥ 0.4 m; h is the slab thickness (cm); P is the axle load (KN).

## 4. Conclusions

In this paper, the impact of void parameters on concrete slab stress is investigated, which is supported by numerical simulation. The location, shape, and size of voids underneath slabs are indicated to have a significant effect on panel stresses. Finally, a function is established to calculate the maximum tensile stress of the concrete slab. The major findings are summarized as follows:

1. C3D8I is discussed as one better element type for its contribution has better accuracy and more convenient practicability. A finite element analysis model considering void morphology is established to analyze the influence of void position, void size and void depth on the stress of concrete slab. The accuracy of the FEA model is verified.
2. The slab stress will increase dramatically when a void area occurs at the slab edge. Nevertheless, slab stress does not change significantly as the void size increases. This may be caused by the change in the stress mode of the concrete slab. The stress mode of the concrete slab in the void area is similar to the bending of the beam supported on three sides, in which the tensile stress at the bottom of the beam is not sensitive to the length of the beam (void area). The variation of the length–width ratio has little effect on the slab stress.
3. Impact of void size and void depth at the slab corner on the slab stress are similar. With the increase in both, the stress at the bottom of the slab decreases slightly and the stress at the top of the slab increases rapidly. When the void size is greater than 0.4 m and the void depth is greater than 0.4 mm, the stress at the top of the slab exceed that at the bottom.
4. A function is established to calculate the maximum tensile stress of the concrete slab. The function takes into account the void size, the slab thickness and the vehicle load. The reliability of the function was verified by comparing the error between the calculated and simulated results.

In future work, the functions of maximum tensile stress can be verified through field measurement and engineering practices. In order to study void better, dynamic loads and joints should be considered. The maximum tensile stress can be used to evaluate the occurrence of longitudinal cracks.

## Figures and Tables

**Table 1:** The characteristics of the 3D finite model.

Layer	Layer Properties	Values
Portland Cement Concrete layer	Slab length	4 m
	Slab width	3 m
	Slab thickness	24–36 cm
	Elastic modulus	30,000 MPa
	Poisson ratio	0.15
Cement and fly-ash stabilized macadam base course layer	Base length	6 m
	Base width	5 m
	Base thickness	18 cm
	Elastic modulus	2000 MPa
	Poisson ratio	0.25
Subgrade	Modulus of subgrade reaction (k)	0.12 MPa/mm

**Table 2:** The results of the convergence analysis.

Mesh Size (h/e)	Element Type				
	C3D8	C3D8R	C3D8I	C3D20	C3D20R
2	1.217	0.926	1.379	1.639	1.571
4	1.394	1.183	1.544	1.637	1.615
6	1.473	1.31	1.589	1.631	1.622

**Table 3:** Comparison between simulation results and function calculation results.

Void Size (m)	Slab Thickness (cm)								
	26			30			34		
	Model	Function	Error	Model	Function	Error	Model	Function	Error
0.6	1.539	1.559	1.30%	1.165	1.169	0.34%	0.911	0.911	0.00%
0.7	1.863	1.859	0.21%	1.385	1.379	0.43%	1.07	1.059	1.03%
0.8	2.198	2.202	0.18%	1.629	1.623	0.37%	1.252	1.232	1.60%
0.9	2.548	2.588	1.57%	1.889	1.902	0.69%	1.453	1.432	1.45%
1	2.991	3.018	0.90%	2.213	2.216	0.14%	1.697	1.657	2.36%
